# Emergency Removal of a Proximal Tracheal Foreign Body by Tracheotomy in a Dog and a Cat

**DOI:** 10.1155/2023/6478643

**Published:** 2023-09-16

**Authors:** Teruo Itoh, Atsuko Kojimoto, Hiroki Shii

**Affiliations:** ^1^Aoba Animal Hospital, 92-1, Aoba-cho, Miyazaki 880-0842, Japan; ^2^Division of Animal Medical Research, Hassen-kai, 2-27 Onozaki, Saito, Miyazaki 881-0012, Japan

## Abstract

There have been few reports of emergency cases of proximal tracheal foreign bodies in dogs and cats. Here, we report a dog and a cat that underwent an emergency tracheotomy for a foreign body in the proximal trachea. Case 1 was a dog with respiratory arrest caused by a large stone in the proximal trachea. The stone was immediately removed via tracheotomy without anesthesia. After intubation and ventilation under anesthesia, hypoxia persisted but improved after aspiration of 100 mL of bloody fluid from the lower trachea. Case 2 was a cat with dyspnea because of a proximal tracheal stone and increased radiopacity in the right lung. The stone was removed via tracheotomy after mask induction of anesthesia, followed by intubation and incision closure. Radiographs immediately after extubation showed worsened right lung atelectasis, alleviated by reintubation and positive pressure ventilation. Both patients recovered completely after surgery. An emergency tracheotomy may be indicated for a large foreign body in the proximal trachea. Additionally, concurrent conditions in the lower respiratory tract should be addressed.

## 1. Introduction

Tracheal foreign body (FB) is a rare disease in dogs and cats [1–3]. Most reported cases had small FBs, such as grass, awns, twigs, seeds, or mineral materials (e.g., stone or tooth) [4–6]. These FBs were commonly found near the carina, with a history of coughing or breathing problems for several days, and were not necessarily an emergency condition [4–6]. For distal tracheal FBs, removal through the mouth with endoscopy [4, 5] or a balloon catheter [7, 8] is preferred, and if these are not feasible, thoracotomy is performed [9–11].

Because a large FB lodged in the proximal trachea can lead to severe or complete lumen obstruction and sudden death, such patients are rarely admitted to the hospital [1, 3, 12]. There is little information on the treatment of proximal tracheal FBs [13, 14]. Here, we report a dog and a cat, each with a large stone lodged in the proximal trachea causing near-complete lumen obstruction, successfully treated by emergency tracheotomy.

## 2. Case Presentation

### 2.1. Case 1

**Figure 1:**
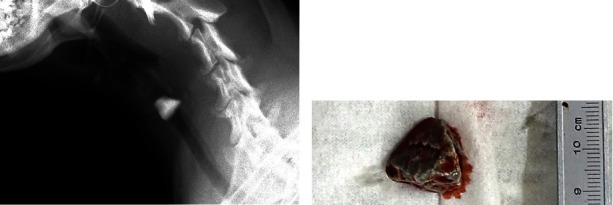
A large foreign body (angular stone) in the proximal trachea with near-complete lumen obstruction in case 1. Right lateral radiographs of the cervical area before surgery (a) and gross appearance of the removed stone (b).

A 12-month-old male golden retriever, weighing 29 kg, presented to our private hospital with dyspnea. The dog was healthy in the morning and was seen playing in the yard, but in the afternoon (5 hours before the visit to our hospital), his breathing became labored and worsened severely 1 hour before the visit. On clinical examination, the dog was dyspneic with a body temperature of 39.1°C and a heart rate of 150 beats per minute. No coughing was heard during the examination. Radiographs showed a radiopaque FB (stone) occupying the proximal tracheal lumen (Figure 1(a)). Blood tests showed mild elevations of aspartate aminotransferase (170 IU/L) and C-reactive protein (1.4 mg/dL) and marked elevation of creatine kinase (> 2000 IU/L).

Inhalational oxygen via a face mask was started with the dog in the sitting position; however, after 30 minutes, he began to refuse the mask intensely, exhibited intermittent panting, and developed respiratory arrest. The ventral neck was immediately shaved and disinfected, and an angular stone (Figure 1(b)) was removed by emergency tracheotomy. A tracheal tube was inserted through the incision, and assisted ventilation was started. Less than 5 minutes elapsed from the respiratory arrest to the tracheal tube insertion. After intubation, the patient showed spontaneous breathing and movement, and then inhalational anesthesia with isoflurane was started. An intravenous catheter was placed for drip infusion. However, even under assisted ventilation, oxygen saturation (SpO2) remained at approximately 90%. Therefore, the tracheal tube was removed and examined, revealing a large amount of bloody fluid. Another tracheal tube was inserted, and a suction device was applied intermittently to the tube opening to aspirate the bloody fluid. The patient was then reintubated through the oral cavity, and the surgical wound was closed.

**Figure 2:**
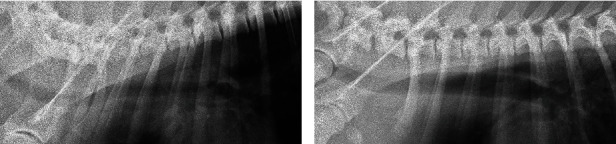
Right lateral radiographs of the thoracic trachea before surgery (a) and after the suction of 100 mL of tracheal fluid (b) in case 1.

Under spontaneous breathing before waking from anesthesia, the SpO2 dropped below 85% when the oxygen delivery tube was detached, leaving the patient on room air. Therefore, anesthesia was prolonged, and suctioning of bloody fluid from the tube was repeated, with changes in the dog's positioning. A total of 100 mL of bloody fluid was aspirated. Subsequent thoracic radiography revealed minimal abnormalities in the lung field, with a clearer intratracheal space than that before surgery (Figure 2). Anesthesia was terminated when the SpO2 on room air reached 88%, and the dog was extubated 45 minutes after surgery. When the dog began to raise his head, the SpO2 was maintained at 95% without an oxygen mask. To prevent bacterial infection to the injured trachea, 18 mg/kg of cephalexin (Larixin®; Fujifilm Co., Ltd., Tokyo, Japan) was prescribed twice a day for 10 days postoperatively. The dog was discharged the next day and recovered uneventfully after that.

### 2.2. Case 2

**Figure 3:**
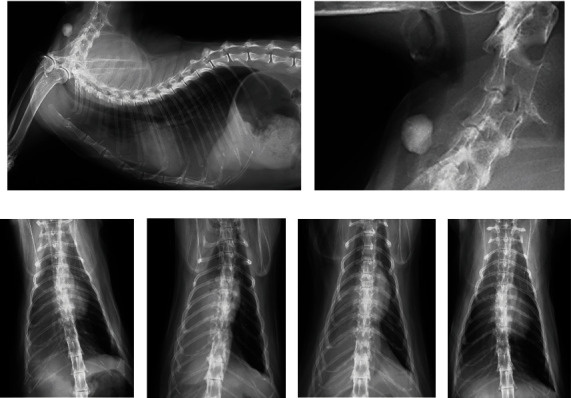
Radiographs of case 2 with a large foreign body (roundish stone) in the proximal trachea. Right lateral cervicothoracic view before surgery reveals increased opacity in the ventral lung field (a) and the stone causing near-complete lumen obstruction (b). Ventrodorsal views demonstrate changes in the right lung opacity before surgery (c), after the first extubation (d), after the positive pressure ventilation before the second extubation (e), and 12 days after surgery (f).

A spayed female, a 6-year-old Japanese domestic cat weighing 4.5 kg, presented with dyspnea after a fight with another cat on the veranda the night before. The cat had not been coughing. Physical examination revealed a body temperature of 38.1°C, heart rate of 175 beats per minute, and respiratory rate of 50 breaths per minute with deep, laboured breathing. Radiography revealed a radiopaque FB (a stone) in the proximal trachea (Figures 3(a) and 3(b)) and slightly increased opacity with alveolar pattern in the ventral area of the right middle and caudal lung lobes (Figures 3(a) and 3(c)). Because aspiration pneumonia associated with the tracheal FB was suspected, emergency surgery was performed.

After subcutaneous administration of 0.05 mg/kg of atropine sulfate, an intravenous catheter was placed. Fifteen minutes later, oxygen was administered by face mask for 3 minutes, followed by mask induction with isoflurane, during which the surgical field was shaved and disinfected. Immediately after induction, the stone was removed by tracheotomy, followed by intubation via the oral cavity. The surgical wound was closed under spontaneous ventilation. Radiographs obtained after extubation revealed worse right lung atelectasis compared with before surgery (Figure 3(d)). After the intravenous administration of propofol, reintubation and positive pressure ventilation with a positive end-expiratory pressure of 6 cm H2O were performed for approximately three minutes, resulting in a reduction in atelectasis (Figure 3(e)). The cat was kept in an oxygen cage overnight and was hospitalized for 5 days to monitor its respiratory condition. To prevent bacterial infection to the injured trachea and atelectatic lung, 8 mg/kg of cefovecin sodium (Convenia®; Zoetis Japan Co., Ltd., Tokyo, Japan) was injected subcutaneously. Additionally, 4.8 mg/kg of enrofloxacin (Baytril®; Elanco Japan Co., Ltd., Tokyo, Japan) was prescribed daily until suture removal 12 days later. The atelectasis had improved (Figure 3(f)), and the cat continued to recover uneventfully after that.

### 2.3. Tracheotomy Procedures

**Figure 4:**
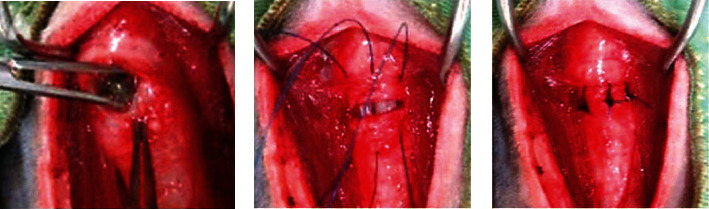
Intraoperative gross view of case 2. After the stone was removed from the tracheotomy site (a), 3-0 absorbable monofilament sutures were passed through the proximal and distal tracheal rings (b), and then tied in a simple interrupted pattern (c).

In both cases, the patients were placed in dorsal recumbency, and the obstructed trachea was approached via a ventral midline incision with the spreading of the sternohyoid muscle. A transverse incision was made between the tracheal rings over the FB, measuring approximately one-third of the tracheal circumference. The intratracheal stone was grasped with mosquito forceps and removed while the incision was dilated using the forceps (Figure 4(a)). The incision was closed with 3-0 glyconate monofilament suture (Monosyn®; B Braun, Rubi, Spain) passed through the proximal and distal tracheal rings (Figure 4(b)) in a simple interrupted suture pattern (Figure 4(c)). After lavaging the operative field with saline, bupivacaine (Marcaine®; Sandpharma Co., Ltd., Tokyo, Japan) (5 mg for case 1 and 1.25 mg for case 2) was applied topically to the surgical wound, and the sternohyoid muscle was sutured with a simple interrupted pattern, followed by subcutaneous and skin closure in accordance with routine procedures. Bacterial cultures of the endotracheal fluid were not performed; empirical antimicrobial therapy was given postoperatively.

## 3. Discussion

Many tracheal FBs are expelled by coughing [1], but some reach the distal trachea or bronchi and remain there, causing persistent coughing and respiratory problems [3]. In humans, complete tracheal lumen obstruction prevents both coughing and breathing, leading to cardiopulmonary arrest within 2-3 minutes if untreated [15]. The FBs in our cases were likely too large for expulsion by coughing or descent into the distal trachea, causing near-complete obstruction. In dogs and cats, most reported cases were FBs in the distal trachea or bronchi [4–12]. Only a few cases of proximal tracheal FBs have been reported; a small segment of pine cone was diagnosed at a chronic stage and removed endoscopically [13], and a large roundish stone was urgently removed by tracheotomy [14]. The cases reported here were also rescued by tracheotomy, although there were concurrent conditions in the lower respiratory tract, and case 1 had more severe respiratory distress than that in a similar case [14].

The Heimlich maneuver has been described as an initial approach for tracheal FBs, in which the animal is swung upside down, or strong pressure is applied to the abdomen [16, 17]. If this method fails, tracheal FBs are removed from the mouth using an endoscope [5, 6], a balloon catheter [7, 8], or fluoroscopically guided forceps [4]. Surgery is generally considered a final option [1–3, 12] except for cases with pneumothorax, empyema, or lung consolidation, for which surgery is selected initially [4, 5]. In light of these general treatment steps, in our cases, the large size of the FBs suggested that the FBs would not easily move and pass through the larynx even with the animal positioned upside down. In endoscopic removal, respiratory management and rapid removal difficulties may be problematic [1]. For these reasons, we planned a cervical tracheotomy initially. As a result, the FB was easily removed immediately after respiratory arrest (case 1) or mask induction of anesthesia (case 2), suggesting the practical utility of tracheotomy in these emergency cases.

While sedation is recommended for anxious, dyspneic animals [18], neither of our cases required it as both were calm during oxygen inhalation. Case 1's aversion to the face mask, observed just prior to respiratory arrest, was likely a result of severe ventilatory failure. For case 2, an anticholinergic drug was administered to prevent bradycardia and tracheal secretion, and no injectable anesthetic was needed due to the smooth acceptance of mask induction. Regardless of premedication or induction method, it appears that prompt tracheostomy is crucial for survival once the patient becomes unconscious. While tracheal incisions can either be left to heal by second intention or sutured using a simple interrupted pattern [18], the latter method was chosen for tracheal FB cases [14, 19, 20].

In humans, tracheal FBs often result in complications like excessive secretion or bleeding, making post-removal suctioning essential [21, 22]. Case 1 had a large amount of bloody fluid in the trachea below the FB. The large, angular stone in this case may have irritated the tracheal wall, leading to these complications. Additionally, expulsion of that fluid by coughing may have been inhibited by near-complete obstruction [15]. Considering that the FB became lodged >5 hours before the dog's presentation and respiratory effort worsened 1 hour before presentation, the subsequent respiratory arrest might have been due mainly to fluid accumulation rather than to direct obstruction from the FB. In similar situations, the suction of fluid from the distal trachea as well as FB removal may be necessary to rescue the patient.

Pneumonia and atelectasis are also common complications in humans with a tracheal FB [21, 22]. Aspiration pneumonia in dogs and cats tends to occur in the ventral area of the right lung due to its gravity-dependent nature [23, 24]. In case 2, the tracheal fluid inflow may have contributed to the right ventral lung lesion. Postoperative worsening atelectasis may have been caused by anesthesia or surgery because it could be significantly alleviated by positive pressure ventilation after reintubation. Postoperative pulmonary complications, including atelectasis, in humans are known to progress immediately after induction of anesthesia, with preoperative hypoxia, respiratory disease, or emergency surgery as risk factors [25]. Because the area of atelectasis is susceptible to bacterial infection [25–27], it is recommended to reexpand the collapsed lung by positive pressure ventilation during anesthesia [25], or after FB removal [21, 22] in humans. However, there are limited reports on this topic in veterinary literature [27], so the validity and necessity of positive pressure ventilation and antimicrobial therapy in similar cases warrant further investigation.

Large FBs in the proximal trachea in dogs and cats are extremely rare to veterinary practice and can be fatal due to severe or complete airway obstruction. The two cases reported here suggest that emergency tracheotomy may be indicated for a proximal tracheal FB with near-complete obstruction. Additionally, concurrent conditions in the lower respiratory tract should be considered and treated for full recovery of the patient.

## Data Availability

The data used to support the findings of this study are included in the article.
